# Music Training, Cognition, and Personality

**DOI:** 10.3389/fpsyg.2013.00222

**Published:** 2013-04-30

**Authors:** Kathleen A. Corrigall, E. Glenn Schellenberg, Nicole M. Misura

**Affiliations:** ^1^Department of Psychology, University of Toronto MississaugaMisissauga, ON, Canada

**Keywords:** music training, music lessons, cognition, personality, individual differences

## Abstract

Although most studies that examined associations between music training and cognitive abilities had correlational designs, the prevailing bias is that music training causes improvements in cognition. It is also possible, however, that high-functioning children are more likely than other children to take music lessons, and that they also differ in personality. We asked whether individual differences in cognition and personality predict who takes music lessons and for how long. The participants were 118 adults (Study 1) and 167 10- to 12-year-old children (Study 2). We collected demographic information and measured cognitive ability and the Big Five personality dimensions. As in previous research, cognitive ability was associated with musical involvement even when demographic variables were controlled statistically. Novel findings indicated that personality was associated with musical involvement when demographics *and* cognitive ability were held constant, and that openness-to-experience was the personality dimension with the best predictive power. These findings reveal that: (1) individual differences influence who takes music lessons and for how long, (2) personality variables are at least as good as cognitive variables at predicting music training, and (3) future correlational studies of links between music training and non-musical ability should account for individual differences in personality.

## Introduction

How do individuals who take music lessons differ from other individuals? In the present investigation, we examined whether duration of music training is associated with the “Big Five” personality dimensions (McCrae and Costa, 1987), the dominant framework for contemporary research on individual differences in personality (John et al., 2008). Much research on associations between music training and non-musical abilities has focused on cognitive skills because such associations are relevant to issues that are central to cognitive science, including modularity (Peretz, 2012), plasticity (Münte et al., 2002), and transfer (Hannon and Trainor, 2007). There is much evidence of lower-level associations between music training, motor skills, and listening abilities (Herholz and Zatorre, 2012), including those related to speech perception (Kraus and Chandrasekaran, 2010; Besson et al., 2011; Strait and Kraus, 2011). Our focus here, however, was on far-rather than near-transfer effects, specifically associations between music training and non-musical cognitive abilities that are less dependent on analytical listening skills or speech perception.

Recent reviews (Costa-Giomi, 2012; Schellenberg and Weiss, 2012) confirm that in addition to being good listeners, musically trained individuals exhibit enhanced performance on tests of verbal abilities, including vocabulary, phonological awareness, reading, and spelling. Music training is also associated positively with performance on tests of spatial abilities and non-verbal reasoning. Because these associations extend across different cognitive domains, they implicate domain-general processes. Indeed, even after accounting for demographic variables, music training is associated positively with performance on tests of auditory *and* visual memory (Jakobson et al., 2008; Degé et al., 2011b), and with IQ (Schellenberg, 2006, 2011a,b; Schellenberg and Mankarious, 2012).

Although most of the associations between music training and cognitive abilities were observed in correlational studies^1^, the prevailing view is that music lessons enhance cognitive abilities, a consequence of inferring causation from correlation. In one widely cited example (Chan et al., 1998), the authors concluded that “music training improves verbal memory” (p. 128) on the basis of comparisons of female college students with or without music training. More recently, after testing bilinguals, musicians, and monolingual non-musicians, the authors concluded that “extended musical experience enhances executive control on a non-verbal spatial task” (Bialystok and DePape, 2009, p. 565). These conclusions tacitly assume random assignment to music lessons even though music training in childhood is associated positively with involvement in *non-musical* extra-curricular activities and with socio-economic variables such as parents’ education and family income (Orsmond and Miller, 1999; Schellenberg, 2006, 2011a; Schellenberg and Mankarious, 2012). Positive correlations between non-musical abilities and duration of music training (or age at which music training began) are similarly interpreted as evidence that music training causes these associations (e.g., Ho et al., 2003; George and Coch, 2011). This interpretation ignores the possibility that high-functioning individuals are more likely than others to begin music training early and to take music lessons for many years.

In some cases, however, music interventions in childhood with random assignment cause improvements in language-related abilities, whether the interventions are long-term (9 months or more) standard pedagogical approaches with children over 8 years of age (Moreno et al., 2009; Chobert et al., 2012; François et al., 2012), or shorter-term (5 months or less) programs designed specifically to develop the listening skills of preschoolers (Degé and Schwarzer, 2011; Moreno et al., 2011a,b). These interventions lead to behavioral and/or electrophysiological improvements in phonological awareness (Degé and Schwarzer, 2011), discriminating syllables that vary in duration or voice-onset time (Chobert et al., 2012), and remembering nonsense words (François et al., 2012). More importantly, positive influences extend from speech perception to vocabulary (Moreno et al., 2011a), associating visual symbols with words (Moreno et al., 2011b), and reading irregularly spelled words (Moreno et al., 2009). In short, interventions that improve music-listening skills are accompanied by improvements in speech perception, which, in turn, enhance some aspects of language processing.

In one experimental study (Costa-Giomi, 1999), fourth-graders from low-SES families were assigned to 3 years of individual piano lessons or no lessons. At the beginning and end of the study, the two groups did not differ in quantitative, verbal, or spatial abilities, although there were some small benefits for the piano group in the interim. In another experimental study (Schellenberg, 2004), first-graders were assigned randomly to 1 year of music or drama lessons taught in small groups, or no lessons. Pre- to post-test increases in IQ were greater (by approximately three points, or 1/5 of a SD) for the children taking music lessons compared to other children. Random assignment necessitated providing free lessons, and practicing between lessons was minimal (10–15 min/week). In other words, although the design allowed for causal inferences, the training differed substantially from the norm, when parents pay for their children to study music.

Regardless, small and intermittent causal effects cannot account for the large cognitive differences between groups that have been reported in correlational studies that compare pre-existing individuals who vary in music training. In one study, for example, children with music training had IQs 10 points (2/3 of a SD) higher than their untrained counterparts (Schellenberg, 2011a). In another study, the difference was 15 points (1 SD; Schellenberg and Mankarious, 2012). Considering that interventions designed specifically to improve cognitive abilities (e.g., Head Start) achieve only modest success during the intervention (effect sizes around 0.20) and much smaller levels of success after the intervention (Love et al., 2013), the available data are best interpreted as showing that high-functioning children are more likely than other children to take music lessons, which may enlarge their pre-existing cognitive advantages. Moreover, because general cognitive ability is relatively stable across the lifespan (Deary et al., 2009), pre-existing differences are also likely to account for associations between music training in childhood and/or adolescence and subsequent cognitive performance in adulthood (Schellenberg, 2006, 2011b).

One of the most intriguing findings to date is that music training in childhood predicts academic achievement even when IQ is held constant (Schellenberg, 2006). In other words, musically trained children are particularly good students, which points to individual differences in non-cognitive abilities or in cognitive abilities other than IQ. Children who take music lessons may have relatively high levels of curiosity, motivation, persistence, concentration, selective attention, self-discipline, and organization. These factors could influence their academic success, their performance on a wide variety of cognitive tasks, and the likelihood that they pursue and continue taking music lessons.

What general constructs that can be measured reliably, besides IQ, might distinguish musically trained from untrained individuals? Although some scholars have suggested a role for executive functions (Hannon and Trainor, 2007; Schellenberg and Peretz, 2008), the results are equivocal in this regard, with associations evident for some measures of executive function but not for others (Bialystok and DePape, 2009; Degé et al., 2011a; Moreno et al., 2011a; Schellenberg, 2011a). Examinations of social skills reveal that drama lessons cause improvement but music lessons do not (Schellenberg, 2004), and that piano lessons (Costa-Giomi, 2004) and music-enrichment classes (Portowitz et al., 2009) are not associated with improvements in self-esteem. Comparable null or inconsistent findings emerge when considering associations between music training and emotional intelligence in adulthood (Trimmer and Cuddy, 2008; Schellenberg, 2011b) or emotion comprehension in childhood (Schellenberg and Mankarious, 2012).

We hypothesized that individual differences in two of the Big Five personality dimensions influence the likelihood that children pursue music training, particularly for extended periods of time. Specifically, learning to play a musical instrument could be facilitated by *conscientiousness*, which involves self-discipline, organization, and achievement-orientation, and/or by *openness-to-experience*, which describes the tendency to have an active imagination, to appreciate the arts and literature, to prefer change and variety over routine, and to be intellectually curious. Such associations could, in turn, help to explain links between music training and cognitive abilities. For example, conscientiousness is associated with academic achievement (Dollinger and Orf, 1991; Furnham et al., 2003; De Fruyt et al., 2008), and this association remains significant even when cognitive abilities are held constant (Bratko et al., 2006). Similarly, openness-to-experience is associated with intelligence (McCrae, 1993; Harris, 2004) *and* academic achievement (Dollinger and Orf, 1991; John et al., 2008).

To date, few studies have examined the possibility of associations between music training and personality, and most of these focused on differences between adult musicians and non-musicians (Kemp, 1996), who do not always exhibit cognitive differences like those found between individuals with or without music lessons (Brandler and Rammsayer, 2003; Bialystok and DePape, 2009; Schellenberg and Moreno, 2010). In other words, comparing professional musicians to equally professional non-musicians is not the same as comparing children with or without music training, very few of whom become professional musicians. Moreover, much of the relevant research was conducted before the emergence of the Big Five taxonomy. These earlier findings revealed that musicians are relatively introverted, independent, sensitive, and anxious (Kemp, 1996). Although undergraduate music students exhibit conscientious-like traits (Marchant-Haycox and Wilson, 1992; Kemp, 1996), composers and rock musicians may actually be *less* conscientious than the general population (Kemp, 1996; Gillespie and Myors, 2000). Compared to non-musicians, musicians tend to be more creative, imaginative, and interested in change, characteristics that are indicators of openness-to-experience (Kemp, 1996; Gibson et al., 2009). In any event, no study to date has examined whether Big Five personality traits are associated with duration of music training, either in childhood or in adulthood, or compared the ability of cognitive and personality variables to predict music training.

Our research questions motivated the use of a correlational design because a true experiment would not enable us to examine whether personality and cognitive variables influence the likelihood of taking music lessons in the first place. Random assignment to music lessons is also plagued by practical, methodological, and generalizability issues. For example, true experiments are often impractical because research funding must be large enough to provide children with music lessons (and even musical instruments) at no cost to participating families (Costa-Giomi, 1999; Schellenberg, 2004). As noted, children who are randomly assigned to “free” music lessons tend to practice their instrument infrequently, in marked contrast to children whose parents are financially invested in music training (Schellenberg, 2004, 2011a). In general, compared to children who take lessons in the real world, children who are assigned randomly to music lessons are likely to be less interested, motivated, and invested in their lessons. Differential attrition among groups receiving music lessons, other lessons, or no lessons (Schellenberg, 2004) also excludes the possibility of long-term experimental studies (e.g., >1 or 2 years). In short, experimental designs are not optimal for studying associations between music training and cognition or personality.

Our participants were undergraduates (in Study 1) and 10- to 12-year-old children (in Study 2) with varying amounts of music training. Demographic, cognitive, and personality data were collected and used as predictor variables in regression analyses to determine the relative importance of personality and cognitive factors in predicting duration of music training. Unlike most previous research (for reviews see Costa-Giomi, 2012; Schellenberg and Weiss, 2012), duration of training was treated as an *outcome* variable rather than a predictor variable, in line with our view that pre-existing differences influence who takes music lessons. Because there is a clear genetic component to general cognitive abilities (Deary et al., 2006) and to personality (Matthews et al., 2003), individual differences in these areas are unlikely to be solely a consequence of an environmental factor such as music training.

We expected that duration of training would be associated with conscientiousness and openness-to-experience even when demographic and cognitive variables were held constant, but we had no predictions about the other personality variables from the Big Five (i.e., agreeableness, extraversion, neuroticism). A secondary prediction was that conscientiousness and/or openness-to-experience would help to explain why musically trained children perform better in school than one would expect from their IQ scores.

## Study 1

In Study 1, we examined associations between duration of playing music regularly and demographic, cognitive, and personality variables in a large sample of undergraduate students. Duration of playing music regularly, which varied widely, was used as the outcome variable (as in Schellenberg, 2006) because it reflected formal training as well as interest in music.

### Materials and methods

#### Participants

The adult sample comprised 118 undergraduates (78 women, 40 men, mean age 20 years) enrolled at a suburban campus in the greater Toronto area, who received partial course credit or token remuneration for their participation.

#### Outcome variable

The undergraduates had, on average, 5.0 cumulative years of private music lessons (SD = 5.5) and 6.4 years of playing music regularly (i.e., private lessons + additional playing, SD = 7.6). On average, those with lessons had discontinued taking them 4.3 years ago (SD = 3.5), and discontinued regular playing 3.2 years ago (SD = 3.3). Years of lessons and playing regularly were highly correlated, *r* = 0.90, *p* < 0.001. In the results that follow, all associations between musical involvement and cognitive and/or personality variables remained evident (but weaker) when duration of lessons was the outcome variable.

#### Predictor variables

As in Schellenberg (2006), annual family income was measured on a nine-point scale (1 = <$25,000, 9 = >$200,000; Canadian dollars, data missing for nine undergraduates). The average family income was between $75,000 and $100,000 per year. Parents’ education was measured on an eight-point scale (1 = did not finish high school, 8 = graduate degree) and averaged across parents. On average, the highest level of education achieved by parents was “some university.”

IQ was measured with the four-subtest version of the Wechsler Abbreviated Scale of Intelligence (WASI; Wechsler, 1999), which is appropriate for participants 6–89 years of age. IQ scores measured with the WASI are highly correlated with IQ as measured by the more comprehensive Wechsler tests (Wechsler, 1999). The average IQ (*M* = 106, SD = 11) was higher than the mean in the US population, *t*(107) = 6.39, *p* < 0.001, as one would expect from a sample of undergraduates, particularly Canadian undergraduates (Canadian norms are slightly higher than US norms).

Personality dimensions were measured with the Big Five Inventory (BFI; John et al., 1991, 2008), a widely used self-report questionnaire that comprises 44 items, with each item rated on a five-point scale. Scores for each personality dimension represent the average rating of the relevant items.

#### Procedure

Participants were tested individually. They were administered the WASI by a trained assistant. They also completed a demographics questionnaire and the BFI.

### Results and discussion

Simple associations among the predictor variables are provided in Table 1. On average, females were more conscientious than males, and participants who came from families with higher incomes also tended to have parents with more education. A positive association between openness-to-experience and IQ confirmed that there was overlap between cognitive and personality variables. Correlations among the Big Five dimensions revealed that higher levels of openness-to-experience tended to be accompanied by higher levels of extraversion, and that extraversion and agreeableness were associated positively with each other and with conscientiousness, but negatively with neuroticism.

**Table 1:** **Correlations among predictor variables in study 1 (undergraduates)**.

	Predictor variables	1	2	3	4	5	6	7	8	9	10
1	Age	–	0.02	0.02	0.06	0.03	0.20*	0.14	−0.03	0.11	0.05
2	Gender		–	0.15	0.00	0.05	0.02	−0.25**	−0.03	−0.14	−0.16
3	Family income			–	0.24*	0.11	0.20*	0.05	0.09	−0.04	−0.01
4	Parents’ education				–	0.04	0.15	0.02	0.07	0.16	−0.13
5	IQ					–	0.29**	−0.04	−0.03	−0.07	−0.02
6	Openness						–	0.15	0.26**	−0.02	0.00
7	Conscientiousness							–	0.31**	0.40**	−0.12
8	Extraversion								–	0.20*	−0.26**
9	Agreeableness									–	−0.29**
10	Neuroticism										–

*p < 0.05, **p < 0.01.

**Figure 1:**
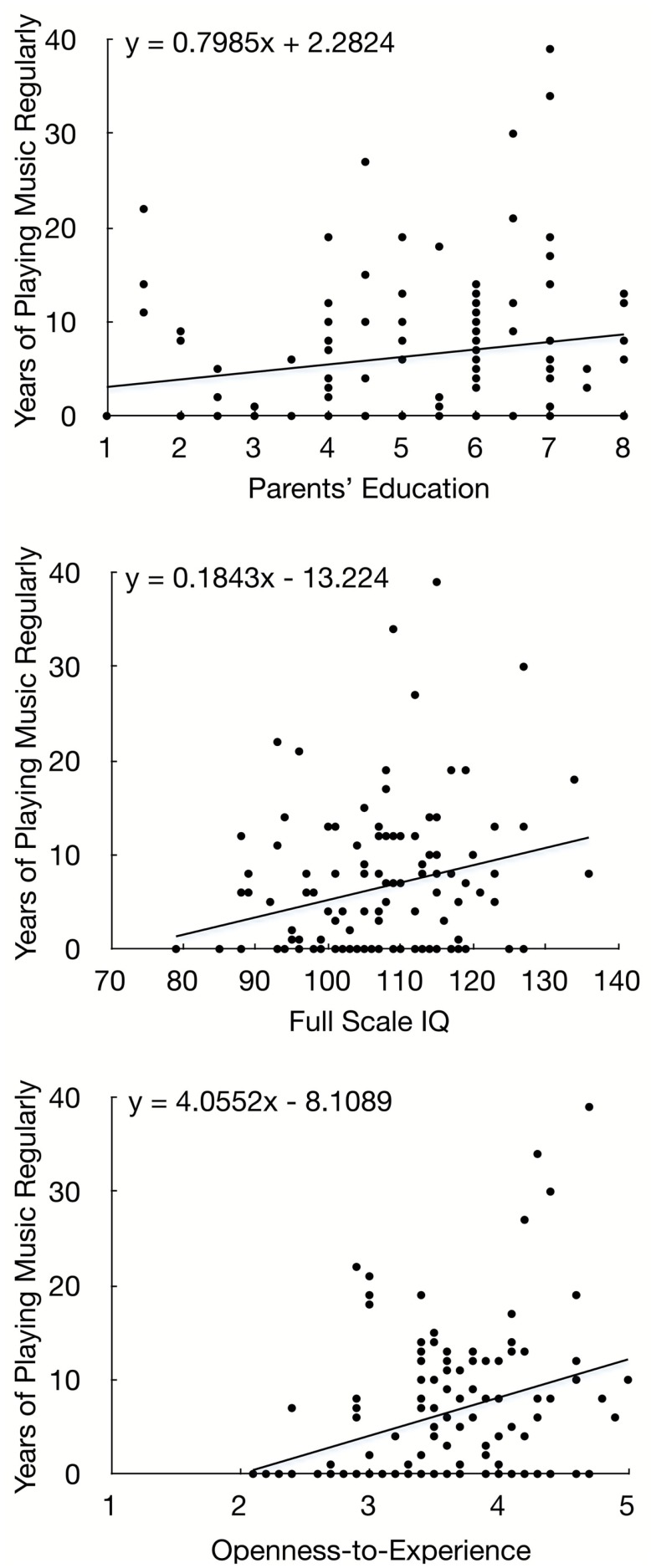
**Study 1 (undergraduates): scatterplots of significant simple associations between predictor variables (*X*-axis) and playing music regularly (*Y*-axis)**.

Tests of simple associations between the predictor variables and the outcome variable confirmed that duration of playing music regularly was correlated with demographic, cognitive, and personality variables. Specifically, undergraduates with a longer history of playing music regularly tended to have more highly educated parents, *r* = 0.19, *N* = 118, *p* = 0.041, higher IQs, *r* = 0.26, *N* = 118, *p* = 0.004, and higher levels of openness-to-experience, *r* = 0.32, *N* = 118, *p* < 0.001. Scatterplots are provided in Figure 1. Duration of playing music regularly was not associated with age, gender, family income, or the four other dimensions of the Big Five, *p*s > 0.16.

Further analyses focused solely on predictor variables that had significant associations with playing music regularly, to determine which of these variables would remain significant with the others held constant. On the first step of a hierarchical multiple-regression analysis (see Table 2), we confirmed an association between playing music and IQ even when parents’ education was held constant. Specifically, parents’ education and IQ accounted for 10.2% of the variance in playing music, and both parents’ education and IQ made significant contributions to the model. When openness-to-experience was added on the second step, the variance explained increased by 5.4%, *F*_inc_(1, 114) = 7.39, *p* = 0.008, such that the model now accounted for 15.6% of the variance in playing music regularly. Both IQ and openness-to-experience made significant contributions to the model, but the contribution of parents’ education was only marginal. Thus, IQ was associated with duration of playing music regularly when demographic and personality variables were held constant, and, more importantly, openness-to-experience was associated with duration of playing music when demographics and IQ were held constant. The results also imply that openness-to-experience is at least as good as IQ at predicting duration of playing music (i.e., the partial correlation was slightly larger in the former case compared to the latter, see Table 2).

**Table 2:** **Results from hierarchical multiple regression in study 1 (undergraduates)**.

Predictor	Partial correlation (*pr)*	*p*-Value
Step 1: *R* = 0.32, *F*(2, 115) = 6.51, *p* = 0.002		
Parents’ education	0.19	0.044
IQ	0.26	0.004
Step 2: *R* = 0.40, *F*(3, 114) = 7.04, *p* < 0.001		
Parents’ education	0.16	0.096
IQ	0.19	0.040
Openness-to-experience	0.25	0.008

*Duration of playing music regularly was the outcome variable*.

## Study 2

In Study 2, we examined associations between duration of music training and demographic, cognitive, and personality variables in 10- to 12-year-old children. The child sample allowed us to examine these associations among participants who were more likely than the adults tested in Study 1 to be actively involved in music training at the time of testing.

### Materials and methods

#### Participants

The sample comprised 167 10- to 12-year-olds (82 girls, 85 boys, mean age 11.5 years) from the local community who received a gift certificate for their participation.

#### Outcome variable

The outcome variable was cumulative months of extra-curricular music lessons (i.e., individual or group lessons outside of the regular school curriculum; *M* = 25.7, SD = 32.4). For those with some training (*N* = 108), 57% were still taking lessons at the time of the study.

#### Predictor variables

Family income was measured as in Study 1 (data missing for 3 children), as was parents’ education. The average annual family income was between $100,000 and $125,000, and the average parent had “some university.” Because children with music lessons also tend to be highly involved in other extra-curricular activities (Schellenberg, 2006), we also collected information about duration of involvement in non-musical out-of-school activities. On average, children had 65 cumulative months of involvement in non-musical activities (SD = 45).

IQ was again measured with the WASI. As one would expect from a sample of middle-class Canadian children, the average IQ (*M* = 112, SD = 11) was higher than American norms, *t*(166) = 13.99, *p* < 0.001. The parent was also asked to provide a copy of the child’s most recent report card (data missing for 11 children). Across publicly funded schools in the province of Ontario, report cards are standardized with the same subject areas and grades reported on the same scales. For each child, grades were converted to numbers (maximum = 12), and an average numerical grade was calculated and used in the analyses (*M* = 9.02, SD = 1.25).

To measure children’s personality, self- and parent-reports were collected from children and their parents, respectively, using the BFI as well as the short version of the Inventory of Children’s Individual Differences (ICID-S; Deal et al., 2007). Parents also provided self-reports of their own personality using the BFI. Preliminary analyses revealed that the children’s five personality scores were correlated across the two different scales (BFI, ICID-S), whether they were completed by the children (BFI scores corrected for acquiescence; Soto et al., 2008), *r*s ≥ 0.19, *p*s ≤ 0.013, or the parents, *r*s ≥ 0.60, *ps* < 0.001. Thus, BFI and ICID scores were standardized and averaged separately for the children’s ratings and those provided by their parents (*M*s = 0, SDs = 1). Children’s five self-report personality scores were correlated with the corresponding scores that parents provided about their children, *r*s ≥ 0.33, *p*s < 0.001, but parent-reports were used in the analyses because they were more stable. Although parents’ own personality scores were correlated with the corresponding scores they provided for their children, *r*s ≥ 0.21, *p*s ≤ 0.007, the modest associations confirmed that parents were making a distinction between their own personality and that of their children. None of the parents’ personality variables was correlated significantly with children’s duration of music lessons, *r*s ≤ 0.15, *p*s ≥ 0.058.

#### Procedure

Children were administered the WASI by a trained research assistant. They also completed the BFI and the ICID-S (self-reports). A parent completed a demographics questionnaire, the BFI twice (once as self-report, once pertaining to the child), and the ICID-S as it pertained to the child.

### Results and discussion

Simple associations among the predictor variables are provided in Table 3. On average, older children had a longer history of non-musical out-of-school activities, and females were more conscientious and agreeable than males. Family income, parents’ education, and involvement in non-musical activities were all positively inter-correlated. Children with higher IQs tended to have more highly educated parents, better grades in school, and longer duration of involvement in non-musical activities. IQ and average grade were associated positively with openness-to-experience and conscientiousness, but negatively with neuroticism. IQ was also correlated positively with extraversion. Finally, correlations among personality variables revealed that neuroticism was correlated negatively with the other four dimensions, which were positively inter-correlated with two exceptions: (1) openness-to-experience and agreeableness, and (2) conscientiousness and extraversion.

**Table 3:** **Correlations among predictor variables in study 2 (children)**.

	Predictor variables	1	2	3	4	5	6	7	8	9	10	11	12
1	Age	–	−0.10	0.06	0.06	0.17*	−0.05	0.14	0.06	0.04	0.01	−0.05	0.08
2	Gender		–	0.02	−0.05	0.10	0.11	−0.11	0.08	−0.19*	0.00	−0.20*	0.09
3	Family income			–	0.44**	0.28**	0.11	0.12	−0.02	0.10	0.00	−0.05	0.00
4	Parents’ education				–	0.22**	0.32**	0.35**	0.04	0.18*	−0.07	−0.08	−0.01
5	Non-musical activities					–	0.15*	0.10	0.09	0.05	−0.04	−0.03	0.08
6	IQ						–	0.47**	0.30**	0.17*	0.16*	0.10	−0.18*
7	Average grade							–	0.26**	0.56**	0.02	0.17*	−0.27**
8	Openness								–	0.24*	0.34**	0.08	−0.21**
9	Conscientiousness									–	0.07	0.44**	−0.47**
10	Extraversion										–	0.17*	−0.39**
11	Agreeableness											–	−0.53**
12	Neuroticism												–

*p < 0.05, **p < 0.01.

**Figure 2:**
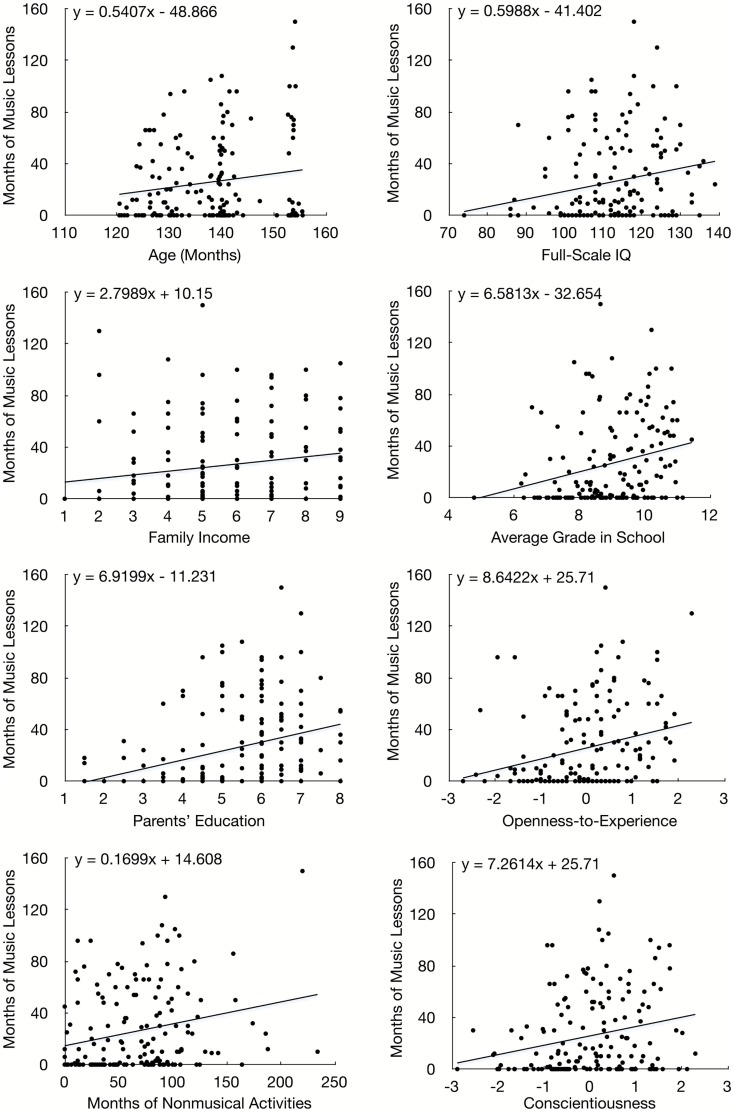
**Study 2 (children): scatterplots of significant simple associations between predictor variables (*X*-axis) and months of music lessons (*Y*-axis)**.

Simple associations between the outcome variable and the predictor variables revealed that children who took music lessons for longer durations tended to be older, *r* = 0.16, *N* = 167, *p* = 0.042, to come from families with higher incomes, *r* = 0.18, *N* = 164, *p* = 0.023, to have parents with more education, *r* = 0.32, *N* = 167, *p* < 0.001, and to have greater involvement in non-musical activities, *r* = 0.24, *N* = 167, *p* = 0.002. They also tended to have higher IQs, *r* = 0.21, *N* = 167, *p* = 0.007, and grades in school, *r* = 0.25, *N* = 156, *p* = 0.002, and to score higher on openness-to-experience, *r* = 0.27, *N* = 167, *p* < 0.001, and conscientiousness, *r* = 0.22, *N* = 167, *p* = 0.004. Scatterplots are provided in Figure 2. These associations confirm that individual differences in demographic, cognitive, and personality variables help to explain if a child will take music lessons and for how long.

As in Study 1, further analyses considered predictor variables that had significant simple associations with music lessons. Because IQ and average grade were highly correlated in the child sample, *r* = 0.47, *N* = 156, *p* < 0.001, as one would expect (Neisser et al., 1996), they were standardized and averaged before consideration of partial associations. The zero-order correlation between this general “cognitive-ability” variable and duration of music training was 0.29, *N* = 156, *p* < 0.001, slightly higher than the simple association between music training and IQ or average grade.

We conducted a hierarchical regression with demographics (age, family income, parents’ education, non-musical activities) and cognitive ability entered on the first step (Table 4), which allowed us to confirm that the association between cognitive ability and music training remained evident when demographic variables were held constant. The regression model accounted for 18.2% of the variance in music training. Significant predictors included parents’ education and cognitive ability. Contributions of age and non-musical activities were marginal. This first step replicated previous results (Schellenberg, 2006, 2011a; Schellenberg and Mankarious, 2012). Children who take music lessons tend to have enhanced cognitive abilities, and this association is evident when age, family income, parents’ education, and involvement in non-musical activities are held constant.

**Table 4:** **Results from hierarchical multiple regression in study 2 (children)**.

Predictor	Partial correlation (*pr)*	*p*-Value
Step 1: *R* = 0.43, *F*(5, 147) = 6.56, *p* < 0.001		
Age	0.14	0.091
Family income	0.04	0.652
Parents’ education	0.19	0.019
Non-musical activities	0.15	0.070
Cognitive ability	0.17	0.035
Step 2: *R* = 0.47, *F*(7, 145) = 5.81, *p* < 0.001		
Age	0.13	0.109
Family income	0.04	0.676
Parents’ education	0.21	0.011
Non-musical activities	0.15	0.066
Cognitive ability	0.06	0.451
Openness-to-experience	0.18	0.028
Conscientiousness	0.09	0.280

*Duration of music lessons was the outcome variable*.

On the second step, we added the two personality variables (openness-to-experience and conscientiousness) to the model. This step tested whether personality helps to explain duration of music lessons with demographics and cognitive abilities held constant. The addition of the two personality variables significantly improved the fit of the model by 3.7%, *F*_inc_(2, 145) = 3.41, *p* = 0.036, with the new model accounting for 21.9% of the variance in duration of music lessons. Parents’ education made the largest contribution followed by openness-to-experience. The contribution of non-musical activities remained marginal. Notably, the variable representing cognitive ability was not even close to significance. In other words, openness-to-experience predicted duration of music training with demographics and cognitive ability held constant, but cognitive ability did *not* predict music training when demographic and personality variables were held constant.

A final analysis asked whether personality variables help to explain why musically trained children are particularly good students. We first confirmed that, as in earlier research (Schellenberg, 2006), music training was associated positively with average grades even when IQ was held constant. Specifically, on the first step of a hierarchical multiple-regression model (Table 5), duration of music training and IQ accounted for 24.1% of the variance in average grade and both IQ and music training made significant contributions to the model. On the second step, we added openness-to-experience and conscientiousness, which improved the fit of the model by 21.0%, *F*_inc_(2, 151) = 28.79, *p* < 0.001, such that the new model accounted for 45.1% of the variance in average grade. Conscientiousness made the largest contribution to the model followed by IQ. In contrast to the previous analysis, openness-to-experience was not a significant contributor, and neither was duration of music training. Thus, individual differences in conscientiousness helped to explain school grades when IQ and music training were held constant, but the link between music training and grades disappeared when personality variables were held constant.

**Table 5:** **Results from hierarchical multiple regression in study 2 (children)**.

Predictor	Partial correlation (*pr)*	*p*-Value
Step 1: *R* = 0.49, *F*(2, 153) = 24.30, *p* < 0.001		
IQ	0.44	<0.001
Music lessons	0.16	0.049
Step 2: *R* = 0.67, *F*(4, 151) = 30.96, *p* < 0.001		
IQ	0.41	<0.001
Music lessons	0.07	0.376
Openness-to-experience	0.00	0.971
Conscientiousness	0.52	<0.001

*Average grade in school was the outcome variable*.

The results of Study 2 reveal that in some instances, personality may be even more important than cognitive abilities at predicting an individual’s likelihood of taking music lessons in the first place, and then persisting at music training for long durations. Our results also suggest that personality factors explain why musically trained children tend to achieve higher grades than their untrained peers even when individual differences in IQ are held constant.

## General Discussion

In groups of undergraduates (Study 1) and 10- to 12-year-old children (Study 2), we examined whether duration of music training was associated with demographic, cognitive, and personality variables, and whether individual differences in personality could shed light on associations between music training and cognitive abilities. Among adults, those with higher IQs, better educated parents, and higher levels of openness-to-experience studied music longer during their childhood and adolescence. Among children, duration of music lessons was associated positively with age, socio-economic status (family income and parents’ education), duration of non-musical extra-curricular activities, IQ, school performance, conscientiousness, and openness-to-experience. The observed associations between musical involvement and multiple individual-difference variables – including those measuring cognitive ability or personality – are virtually impossible to be solely a consequence of music training.

In both studies, personality variables predicted duration of music training even when demographics and cognitive ability were held constant. Among children, moreover, cognitive ability no longer predicted duration of music training when demographic and personality variables were held constant. Note that by collapsing IQ and average grade into a single variable, we actually increased power (i.e., 1 df instead of 2, larger simple association with music lessons). Our results suggest that when predicting who is likely to take music lessons and for how long, individual differences in personality are at least as important as cognitive variables among adults, and even more important among children. They also raise questions about virtually all previously reported correlations between music training and cognitive abilities that failed to account for personality (e.g., Roden et al., 2012). Moreover, despite the oft-cited claim that music training is a good or ideal model for the study of plasticity (Münte et al., 2002; Jäncke, 2009a,b; Herholz and Zatorre, 2012), our findings highlight pre-existing differences between children who take music lessons and those who do not in terms of demographic variables, cognitive abilities, and personality traits. It is nevertheless possible that music training serves as a mediating variable between personality and cognitive abilities, such that personality influences who takes music lessons, which, in turn, enhance cognitive abilities.

In neither sample was musical involvement associated with agreeableness, extraversion, or neuroticism, but no such associations were expected. One somewhat surprising finding was that compared to conscientiousness, openness-to-experience was a better predictor of involvement in musical activities. For example, among adults, conscientiousness was not associated with duration of playing music regularly. Among children, only openness-to-experience contributed unique variance in predicting duration of music training when demographic and cognitive variables were held constant. Nevertheless, relatively high levels of conscientiousness rather than openness helped to explain why musically trained students do better in school than one would predict from their IQs. In short, individuals who choose to take music lessons are primarily those who are interested in learning and experiencing new things, especially in artistic domains, but they do well in school because they have high IQs and because they are particularly hard-working and self-disciplined.

As with IQ, personality variables have a well-defined genetic component, in line with our view that such individual differences are pre-existing. For example, each dimension of the Big Five has a heritability estimate of approximately 0.5 (Bouchard and Loehlin, 2001), similar to heritability estimates for IQ, although the genetic contribution to IQ increases with age because individuals increasingly seek out environments that match their cognitive predispositions (Neisser et al., 1996). Personality shows similar signs of stability as a consequence of genetic factors, with temperament in infancy predicting personality in adulthood (Caspi et al., 2005). As with IQ, niche-building tendencies lead individuals to environments that match their personality, with such environments promoting additional stability in personality over time (Caspi et al., 2005).

In any event, we do not deny a role for the environment in shaping cognitive ability (or personality), which is ultimately a consequence of nature and nurture. Nevertheless, most researchers favor one interpretation over the over. Our parsimonious proposal is that different individuals choose different activities (including music lessons) in contrast to the conventional view that music lessons make individuals different, or that music lessons serve as a mediating variable between pre-existing traits and cognitive functioning. Because far-transfer effects are very infrequent without substantial overlap in: (i) what is being transferred and (ii) the context in which such transfer will occur (Barnett and Ceci, 2002), the burden of proof should rest on those who claim systematic far-transfer effects from music lessons to cognitive abilities.

Although we have no doubt that music lessons change behavior as well as neurological structure and function, the question is whether they do so systematically. In isolated instances, the causal direction seems clear, as when violinists have enlarged cortical representations of the fingers on their left hand (Elbert et al., 1995). Music lessons almost certainly improve listening abilities (Kraus and Chandrasekaran, 2010; Besson et al., 2011; Strait and Kraus, 2011; Herholz and Zatorre, 2012), which enhance speech perception (Degé and Schwarzer, 2011; Chobert et al., 2012; François et al., 2012), which, in turn, enhances some aspects of language processing (Moreno et al., 2009, 2011a,b). Nevertheless, many of the listening tasks that have been used (e.g., detecting pitch changes) resemble those used to measure music aptitude (i.e., natural musical ability; e.g., Wallentin et al., 2010). Individuals with low music aptitude would be unlikely to pursue music lessons, which would guarantee a positive correlation between pre-existing listening abilities and music training even before the training begins. The proposal of a causal link *from* music training *to* listening abilities is also belied by evidence indicating that the association is moderated by motivational state (McAuley et al., 2011).

The idea that a potentially enjoyable activity such as learning to sing or to play a musical instrument could have beneficial side-effects on cognitive functioning is obviously appealing. It is important to remain realistic, however, about the power of music training to alter cognitive abilities. Enthusiasm about plasticity, particularly among neuroscientists (Münte et al., 2002; Jäncke, 2009a,b; Herholz and Zatorre, 2012), must be balanced with an awareness of findings from behavioral genetics, which reveal a genetic component to virtually all behaviors (Bazzett, 2008). Much previous research may have overestimated the effects of music training and underestimated the role of pre-existing differences between children who do and do not take music lessons. Our results implicate a role for personality – in addition to demographics and cognitive abilities – in the decision to take music lessons and in the continuation of such lessons for extended periods. Accordingly, future investigations of associations between music training and non-musical abilities should account for individual differences in personality.

## Conflict of Interest Statement

The authors declare that the research was conducted in the absence of any commercial or financial relationships that could be construed as a potential conflict of interest.

## Footnotes

^1^We use “correlational” to refer to both “correlational” and “quasi-experimental” designs. In correlational designs, music training varies continuously. In quasi-experimental designs, groups of musically trained and untrained individuals are compared. Because there is no random assignment, neither design allows for causal inferences.
